# Unexplained and Unexpected Pediatric Deaths: Forensic Review and New Perspectives

**DOI:** 10.3390/diagnostics13193111

**Published:** 2023-10-02

**Authors:** Isabella Aquila, Matteo Antonio Sacco, Jan Gorniak, Melanie Rouse, Saverio Gualtieri, Fabrizio Cordasco, Alessandro Pasquale Tarallo, Roberto Raffaele, Pietrantonio Ricci

**Affiliations:** 1 Institute of Legal Medicine, Department of Medical and Surgical Sciences, University “Magna Graecia” of Catanzaro, Viale Europa, Loc. Germaneto, 88100 Catanzaro, Italy; matteoantoniosacco@gmail.com (M.A.S.); saveriogualtieri@icloud.com (S.G.); cordasco@unicz.it (F.C.); drtarallomedlegale@gmail.com (A.P.T.); raro81@libero.it (R.R.); ricci@unicz.it (P.R.); 2 Clark County Office of Coroner Medical Examiner, 1704 Pinto Lane, Las Vegas, NV 89106, USA; drquincydo@sbcglobal.net (J.G.); melanie.rouse@clarkcountynv.gov (M.R.)

**Keywords:** unexplained pediatric death, unexpected pediatric death, autopsy in pediatric death, standardization

## Abstract

Unexplained and unexpected pediatric deaths are a major challenge in global healthcare. The role of the forensic pathologist is crucial in determining the cause and manner of death in these cases, although to date, there are many limitations in post-mortem diagnosis. The role of the autopsy and related forensic investigations become a fundamental tool to investigate and give an explanation for an unacceptable event, considering the young age of the victims. From this point of view, even today, it is necessary that this phenomenon is correctly recorded through standardized systems and universally accepted methods. Furthermore, it is essential that scientific research on this topic is increased through the implementation of universally accepted operating protocols recognizing real risk factors in order to prevent such events. The purpose of the study is to offer a review of the state of the art about unexplained pediatric death and, above all, to propose an international reporting platform, extending proper investigations not only to judicial cases but also to all the other cases of unexpected pediatric death.

## 1. Introduction

In 2020, the infant mortality rate in the United States was calculated at 541.9 deaths per 100,000 births [1]. Sudden unexpected infant death (SUID) deaths amount to approximately 3400 per year in the United States alone [2]. A variable rate has been described from country to country in the world. SUID represents the leading cause of postnatal mortality in the world. It is an umbrella category that describes all sudden, unexpected infant deaths—those from known causes and those from unknown causes. This form of death may be associated, after appropriate investigations, with accidental suffocation, sudden infant death syndrome (SIDS), or uncertain circumstances. According to the San Diego definition, SIDS is the sudden unexpected death of an infant <1 year of age, with the onset of the fatal episode apparently occurring during sleep, that remains unexplained after a thorough investigation, including the performance of a complete autopsy and review of the circumstances of death and the clinical history [3]. Sudden unexplained death in childhood (SUDC), on the other hand, is the death of a child over 1 year old that is unexplained despite the analysis of the clinical history, the evaluation of the circumstantial data, and the completion of an autopsy. The age group most affected by SUDC is between 1 and 4 years [4,5].

### 1.1. Risk Factors, Classifications, and Scene Investigations with the Help of Doll Reenactment

Unexplained pediatric deaths are a tragic and complex issue that affects families and communities worldwide. Understanding the risk factors, classifications, and scene investigations associated with these deaths is crucial to preventing and solving such cases. The following sections will explore the common risk factors associated with unexplained pediatric deaths and the role of doll reenactment in assessing these factors, i.e., a doll that is used to reproduce the circumstances under which the baby was found. Additionally, the paper will examine how doll reenactment can be employed to classify unexplained pediatric deaths and the information that can be obtained from scene investigations.

#### 1.1.1. Risk Factors Associated with Unexplained Pediatric Deaths

Pediatric deaths can be caused by various factors, such as unintentional suffocation and SIDS. Research has shown that bed-sharing is involved in the majority of such cases and that the majority of SIDS deaths occur in non-crib sleeping areas. Several specific risk factors have been identified for SIDS, including extrinsic risk factors such as cigarette smoking and drug and alcohol abuse, but also intrinsic factors like premature birth, maternal intrauterine diseases, demography, or sex [6]. Additionally, black infants under the age of three months are more likely to suffer from unintentional suffocation or SIDS [7].

SUDC shares some characteristics with SIDS but also shows important differences. The role of maternal smoking and bed-sharing appears to be minor in SUDC. Precipitating factors of SUDC may be infections or fever. Epileptic seizures are also among the suggested risk factors [8,9]. Moreover, the prevalence of febrile seizures also needs to be more accurately defined, and other risk factors in SUDC cases need to be identified to compare with sudden explained deaths in childhood [10]. It has been observed that in some cases, a febrile seizure may have contributed to or caused death. Additionally, over 75% of SUDC cases show fever or illness symptoms within 48 h of death. While SUDEP, or Sudden Unexpected Death in Epilepsy, is similar to fatalities caused by febrile seizures, it is important to note that cases of febrile seizures are not categorized as SUDEP [10]. Additionally, it is worth mentioning that siblings of children who have experienced sudden unexplained death did not experience sudden deaths themselves. However, rates of febrile seizures were higher among both sudden explained and sudden unexplained deaths compared to the general population.

#### 1.1.2. The Role of Doll Reenactment in Understanding the Risk Factors of Unexplained Pediatric Deaths

Innovations in death scene investigations have led to the introduction of doll reenactment, based on the reproduction of the exact position in which the infant was found at the moment of death, which has proven to be a useful tool in identifying risk factors for unexplained pediatric deaths [11]. By analyzing the modifiable risk factors like bed-sharing, cigarette smoking, and soft bedding, among others, medical examiners have become more cautious in assigning SIDS as the cause of death [11]. For instance, medical examiners may now assign accidental suffocation or strangulation in bed as the cause of death when a risk factor consistent with asphyxia is present [11]. Hence, doll reenactment and other improvements have enabled the detection of possible asphyxia as a risk factor for unexplained pediatric deaths [11]. However, opinions about assigning SIDS as the cause of death in cases where no apparent cause is found during post-mortem investigations vary among medical examiners [11]. Hence, it is crucial to conduct a thorough and accurate death scene investigation by trained personnel, including a scene recreation with a photographed or videotaped doll reenactment, to gain a better understanding of the risk factors for unexplained pediatric deaths [12].

#### 1.1.3. How Can Doll Reenactment Be Used to Assess the Risk Factors Associated with Unexplained Pediatric Deaths?

In some jurisdictions, doll reenactment is used to assess the risk factors associated with unexplained pediatric deaths [13,14,15,16,17,18,19,20]. This technique is used to gain more accurate information regarding the risk factors and develop an understanding of the circumstances that led to the death [14]. Through this approach, modifiable risk factors such as maternal cigarette smoking, bed-sharing, and soft bedding can be identified and taken into consideration [19]. As a result, when a risk factor is consistent with possible asphyxia, medical examiners may assign the cause of death as accidental suffocation or strangulation in bed [17]. Moreover, a death scene investigation form is used to capture the relevant information [14]. It also includes a medical history section to identify potential SUID risk factors [20]. Additionally, a scene investigation is conducted, which involves a doll reenactment [12]. This technique helps visualize the scene, giving the medical examiner an idea of the incident location and the potential SUID risk factors [20]. Although the quality of doll reenactments can vary, it still provides an efficient way to study the risk factors associated with unexplained pediatric deaths [18].

### 1.2. Classifications and Scene Investigations of Unexplained Pediatric Deaths

#### 1.2.1. How Can Doll Reenactment Be Used to Classify Unexplained Pediatric Deaths?

Doll reenactment can be used as a tool to help classify unexplained pediatric deaths. This technique involves photographing or videotaping a scene recreation of an infant’s death [12]. The accuracy of the autopsy report and the resources allocated for the case rely heavily on the accuracy of the scene investigation. Doll reenactment is thus an important part of this investigation, as it helps to determine the cause and manner of the infant’s death [12]. The doll reenactment should be conducted as part of a comprehensive death scene investigation, which should also include body diagrams and a standardized format [12]. Through the use of doll reenactment, forensic investigators can recreate and study the circumstances of the infant’s death more accurately. This helps to classify the death in a more precise manner and can be used to inform the autopsy report. It is also useful for providing closure to the family of the deceased infant.

#### 1.2.2. What Information Can Be Obtained from Scene Investigations of Unexplained Pediatric Deaths?

Scene investigations of unexplained pediatric deaths can provide valuable information [21]. Autopsies of 107 unexplained pediatric deaths revealed that SIDS and SUDI were the primary causes of death [22]. Among the deaths investigated, 32% of SUDI cases showed prodromal symptoms, and an unsafe sleep environment was found in some cases of SIDS [22]. In addition, microscopic findings suggestive of respiratory infection were found in some cases of SIDS. Moreover, ocular hemorrhages can be a valuable source of information about an unexplained pediatric death [21]. In a study conducted by Gilliland et al., it was discovered that 70 out of 169 child deaths that were referred to a medical examiner exhibited retinal hemorrhages [21]. The study also found that ocular hemorrhages are strongly correlated with retinal, peripheral retinal, optic nerve sheath, and intrascleral hemorrhages. However, this study’s design had its limitations and did not provide any information about hemorrhage patterns in children who underwent cardiopulmonary resuscitation (CPR). It is important to keep in mind that retinal hemorrhages in children who underwent CPR cannot be solely attributed to CPR since most cases involved coincident causes such as head trauma [21]. Thus, scene investigations of unexplained pediatric deaths can provide a more comprehensive understanding of the cause of death [21].

#### 1.2.3. How Can Doll Reenactment Help in Understanding the Scene Investigations of Unexplained Pediatric Deaths?

To better understand the investigations surrounding SUID, a study of medical examiners and coroners (ME/Cs) was conducted [22,23]. The results of this study revealed that the classification of sudden infant deaths has a significant impact on our ability to accurately monitor mortality trends and comprehend their underlying causes [16]. Additionally, the reported causes of death were organized into nine primary categories [24]. Surprisingly, SIDS was rarely used as a cause of death classification by ME/C offices [14]. The surveys also indicated that the classification and coding of sudden, unexpected infant deaths remain a challenge. To overcome this issue, ME/Cs were encouraged to utilize scene and doll reenactments [17]. In Salt Lake City, a discrepancy in the classification and coding of SUID in two different racial categories was observed [25]. It was also established that a complete scene investigation using doll reenactment is an essential part of the death investigation process [13]. In addition, understanding genetic predispositions to unexpected death contributes to the significance of doll reenactment [15]. Finally, the surveys showed that the case types that should be investigated and how infant deaths fit into them are still not fully understood [20,21,22,23,24,25,26,27,28]. This is why doll reenactment is so important, as it helps pinpoint the risk factors that may have caused the death. It is also useful in detecting wedging, which is essential in understanding the infant’s death.

The research paper on examining unexplained pediatric deaths sheds light on the risk factors, classifications, and scene investigations involved in SIDS and SUDC. The study highlights that unexplained pediatric deaths can be caused by various factors, including unintentional suffocation and febrile seizures. The prevalence of febrile seizures needs to be accurately defined, and other risk factors in SUDC cases need to be identified to compare with sudden explained deaths in childhood. The paper asserts that bed-sharing is involved in the majority of such cases, and the majority of SIDS deaths occur in non-crib sleeping areas. The research has shown that in some cases, a febrile seizure may have contributed to or caused death. Moreover, the study reveals that black infants under the age of three months are more likely to suffer from unintentional suffocation or SIDS. The research also establishes that a thorough scene investigation with doll reenactment is an essential part of the death investigation process. Doll reenactment is one of the innovations that have been introduced in death scene investigations to understand the risk factors of unexplained pediatric deaths. The study concludes that it is essential to conduct a thorough and accurate death scene investigation by trained personnel to detect possible asphyxia as a risk factor for unexplained pediatric deaths. The paper also emphasizes the need to understand genetic predispositions to unexpected death, contributing to the significance of doll reenactment. The research paper has identified several gaps in the understanding of unexplained pediatric deaths and suggests future directions for research, including accurate classification of sudden unexpected infant deaths in different racial categories. Overall, the study provides valuable insights that can help prevent unexplained pediatric deaths and improve death scene investigations.

### 1.3. Understanding Unexplained Pediatric Death: The Role of Family and Witness Interviews, Medical Records, and Cause-of-Death Analysis

Unexplained pediatric deaths pose a significant challenge for medical professionals and families alike. Understanding the cause of such deaths is crucial for preventing future occurrences and providing closure for families. This research paper aims to explore the role of family and witness interviews, medical records, and cause-of-death analysis in understanding unexplained pediatric deaths. By examining these factors, we can gain a better understanding of the complexities involved in investigating unexplained pediatric deaths and provide insights into potential causes. This paper will examine the significance of each of these factors and how they can be used together to provide a more comprehensive understanding of unexplained pediatric deaths. Ultimately, our research seeks to contribute to the development of improved practices for investigating and preventing unexplained pediatric deaths.

#### 1.3.1. What Role Do Family and Witness Interviews Play in Understanding Unexplained Pediatric Death?

For the purpose of studying SUDEP, it is essential to ascertain cases in detail by interviewing bereaved families and witnesses [7]. During the case ascertainment for SUDEP, any witnessed deaths are identified, and the circumstances surrounding them are examined thoroughly [27]. This provides a much better understanding of the events that occurred prior to the child’s death. It is important to remember that the family and witness interviews provide valuable insights into the circumstances and events that occurred prior to the death of the child. This is an important part of understanding the cause of death in such cases. It is important to note that the family and witness interviews provide a unique opportunity to obtain a more complete understanding of the events that occurred prior to the child’s death [27]. This is why it is important to include a detailed assessment of the family and witness interviews in order to obtain an accurate picture of the events leading up to the death of the child.

#### 1.3.2. How Can Medical Records Provide Insight into the Cause of Death?

Medical records can provide insight into the cause of death, particularly when sudden death occurs with no obvious symptoms. Local Centers for Disease Control and Prevention (CDC) and hospital records can be used to gather information, as well as antiepileptic drug levels, toxicological screens, post-mortem findings, and autopsied deaths [28]. The cause of death can further be understood by examining the past medical history of the deceased, which is usually listed on the death certificate. In addition, a unified autopsy form can be used for quality control, and the first witness can be contacted for information related to the cause of death [28]. Verbal autopsy can also be performed to collect information, as well as to identify cases of sudden cardiac death [28,29,30,31,32]. Furthermore, medical records can be used to gain insight into the scope and nature of sudden death, as well as to identify potential causes of sudden cardiac death [29]. Data collection should include seizure types, premorbid antiepileptic drug levels, general level of compliance, and stressful circumstances preceding death, and more detailed data is needed in order to understand the cause of death [28]. Furthermore, prospective data collection is important to gain insight into the cause of death, while oral autopsy data could provide detailed information [32]. Studies have also shown that an arrhythmic cause could account for approximately 20–30% of sudden unexplained deaths among younger patients and that cardiac genetic mutations contribute to a sizeable proportion of sudden unexplained deaths [28,29,30,31,32].

#### 1.3.3. What Can the Cause-of-Death Analysis Tell Us about Unexplained Pediatric Death?

Cause-of-death analysis can provide invaluable insight into the cause of unexpected pediatric deaths related to epilepsy [30]. Despite the need for further exploration into this area, there is a dearth of prospective studies that have evaluated the causes of mortality in well-characterized cohorts of pediatric patients with epilepsy [30]. Autopsy data is essential for reliably determining the cause of SUDEP in pediatric patients. Post-mortem findings can be assessed to determine the cause of death, and antiepileptic drug levels may be examined to identify possible cases of poor antiepileptic drug (AED) compliance. Additionally, the past medical history of the deceased can be documented on their death certificate and examined to ascertain the cause of death [30]. It is important to note that cause-of-death analysis is only one part of the puzzle when it comes to understanding the cause of pediatric deaths related to epilepsy. Other factors, such as environmental influences, genetic predispositions, and even lifestyle choices, must all be taken into account [30]. Thus, a comprehensive approach to understanding the cause of pediatric deaths related to epilepsy requires a multi-faceted approach [30].

The multi-faceted approach to understanding the cause of pediatric deaths related to epilepsy presented in this research paper highlights the importance of utilizing a variety of resources to gain insight into the events leading up to the child’s death. Family and witness interviews provide valuable contextual information, while medical records and cause-of-death analysis can offer insight into the underlying cause of death. However, it is important to note that cause-of-death analysis alone is not enough to fully understand the circumstances of the child’s death. The limitations of this study include the potential for biases in the recollection of events by family and witnesses, as well as the possibility of incomplete medical records. Future research should focus on prospective data collection and the use of oral autopsy data to provide detailed information on the events leading up to a child’s death. Additionally, the role of cardiac genetic mutations in sudden unexplained deaths among younger patients should be further examined. Ultimately, a comprehensive approach to understanding unexplained pediatric deaths, including a thorough examination of all available resources, is necessary to improve prevention and treatment strategies.

## 2. Materials and Methods

In this non-systematic review of the literature, a search was conducted on the Scopus, Google Scholar, Wos, and Pubmed Ncbi engines by inserting the following keywords: “Unexplained and unexpected Pediatric Deaths AND Risk factors, Unexplained and unexpected Pediatric Deaths AND Classifications, Unexplained and unexpected Pediatric Deaths AND Scene investigation, doll reenactment, autopsy and Unexplained and unexpected Pediatric Deaths, informatic system of UPD”. Over 200 papers were screened by the title, and 123 papers—published between 2013 and 2023—were selected for the abstract analysis. About half of the abstracts were excluded because they were not relevant to the purpose of the literature review. Only the papers relevant to the keywords were selected through operator-dependent selection for full paper reading. In particular, those works that focused on the role of the autopsy in these cases and on the standardization in the autopsy report were read. Also, relevant papers found by reference analysis were selected.

## 3. Results

A total of 25 studies were incorporated into the literature review. The range for the pediatric population examined in this study was until 4 years old.

### 3.1. The Role of the Forensic Pathologist in Unexplained Pediatric Deaths

#### 3.1.1. What Is the Role of the Forensic Pathologist in Determining the Cause of Death in Unexplained Pediatric Deaths?

The review revealed that the role of the forensic pathologist is pivotal in determining the cause of death in unexplained pediatric deaths [33]. Post-mortem CT is not considered sufficient for accurately determining the cause of death in these cases [34]. A consensus classification of unexplained sudden deaths in infants and children has been established to aid forensic pathologists in their investigations. The Radcliffe Congress also provides explicit guidance to death certifiers regarding the use of other diagnoses. The inclusion of “unexplained sudden death in children and adults/MH12” as a diagnostic category is expected to enhance global surveillance of these deaths in children beyond infancy [33]. Molecular autopsies have been utilized to diagnose cardiac channelopathies in post-mortem cases, with pathogenic mutations identified in over one-third of sudden unexplained death cases through molecular autopsies [33]. Forensic pathologists are expected to adhere to rigorous criteria before utilizing “undetermined” or asphyxia categories that diverge from SIDS. Families must be given priority, with clear explanations of the evidence for why the infant passed away. Precise and descriptive terminology is also necessary to ascertain the cause of death in such cases. When assessing a death for asphyxia, the mere existence of environmental risk factors during sleep, without any proof of airway blockage or compression of the chest wall, is not enough to declare the cause of death as asphyxiation. It is imperative that the forensic pathologist relies on concrete and unbiased evidence to reach their conclusions regarding the cause of death. This approach is crucial to maintain the accuracy and reliability of the final determination.

#### 3.1.2. What Are the Limitations of Post-Mortem Diagnosis in These Cases?

In cases of unnatural death, a post-mortem diagnosis can be a valuable tool for determining the cause of death. However, it is important to note that there are limitations to its effectiveness, especially in cases of perinatal and forensic childhood deaths where there may be a lack of specificity in imaging findings. In situations where traditional autopsy methods cannot establish the cause of death, a post-mortem diagnosis may not be as useful. It is also important to consider the implications of negative findings, which may still hold significance for families and clinicians seeking answers about the death. For instance, post-mortem imaging can help identify structural lesions in cases of suspected non-accidental injury or sudden unexpected death in infancy. Nonetheless, imaging very small fetuses can prove to be quite challenging as it requires overcoming the limitations of post-mortem magnetic resonance imaging (PMMR) and other imaging techniques. Addressing these limitations, particularly in regard to imaging very small fetuses, is crucial. Although PMMR and other imaging techniques have their limitations [34,35,36], it is important to acknowledge that there are also limitations in diagnosing post-mortem cases such as terminations of pregnancy, intrauterine death, sudden unexpected infant death, and identification issues [36,37]. A comprehensive review of current issues and challenges in cross-sectional imaging for post-mortem cases is necessary, especially since significant series on post-mortem imaging are not readily available in pediatric and perinatal practice. While scientific evidence for post-mortem imaging is robust in MRI and central nervous system analysis, there is still much work to be done to improve the accuracy of post-mortem diagnosis.

#### 3.1.3. Autopsy and Related Forensic Investigations to Explain These Deaths

Autopsy and related forensic investigations are essential in the investigation of sudden unexplained deaths [38]. Autopsies can help to determine the cause of death, the manner of death, and the time of death [38,39,40,41]. In addition, forensic investigations can also provide information on the use of drugs and help in linking a suspect to the scene of a crime in cases involving child neglect, sexual abuse, and identification of suspects. The primary functions of a forensic pathologist include external and internal examination of the cadaver, analysis of antemortem injuries and post-mortem changes, and recovery of physical evidence. It is also important to investigate the circumstances of death in order to differentiate between natural and unnatural causes of death [38,39,40]. Death scene investigations in cases of SIDS can generate new hypotheses concerning risk factors. The attendance of death scenes will depend on local governance and legislation. In addition, scoring systems have been developed and applied to standardize death scene investigations in some studies. The effects of autopsy and related forensic investigations can be far-reaching, impacting not only the deceased’s family but also various entities such as law enforcement agencies, hospitals, and insurance companies [38,39,40]. Despite advancements in this field, there are still cases that are classified as “unascertained” when the cause and manner of death are not determined after an autopsy. To investigate sudden unexplained deaths, a multidisciplinary translational research team is required, and molecular diagnosis is progressively being utilized in forensics due to the development of genetics. In addition, new genetic technologies may assist in identifying the genetic cause of sudden death, and recognizing an inheritable defect that causes arrhythmogenic syndromes can help family members at risk of sudden death take preventive measures. Ultimately, autopsy and forensic investigations can provide clarity to these deaths, and thanks to modern technologies such as next-generation sequencing (NGS), molecular autopsy may be able to identify the cause of death in a considerable number of unsolved cases. In the literature, Montanari et al. deeply analyzed the apparently hidden reasons for one case of in utero death: toxicological and genetic causes, both able to shed light on the real cause of death [41,42].

#### 3.1.4. Italian Law n. 31-2 February 2006

The Italian Law n.31, “Regulations for Diagnostic Post Mortem Investigation in victims of the Sudden Infant Death Syndrome (SIDS) and Unexpected Fetal Death” is a revolutionary law that introduced fundamental elements in the field of SIDS.

According to the law, all Italian regions must identify reference centers for the examination of children who die suddenly within a year or fetuses without apparent causes after the 25th gestational week. Art. 1 of the law establishes that these infants must be rapidly subjected to post-mortem investigations in authorized centers with the consent of both parents. A family interview should be conducted regarding any risk factors for SIDS [43]. Art. 4 of this law establishes that health authorities must promote awareness campaigns to ensure correct information on problems associated with SIDS and must arrange for multidisciplinary research programs that include anamnestic, laboratory, and histological data in these cases.

### 3.2. Need for Standardized Systems and Protocols

#### Standardized Systems and Universally Accepted Methods to Document Unexplained Pediatric Deaths

In order to achieve a variety of objectives, it is essential to establish standardized systems and universally accepted methods for documenting unexplained pediatric deaths. These systems and methods help to ensure a uniform collection of information for death investigations and autopsies, which can facilitate better sharing of information for prevention purposes [44]. Additionally, they are crucial for correctly classifying the cause and manner of death. It is fundamental to submit the victims of SIDS and sudden intrauterine unexpected death to detailed post-mortem investigations, according to a protocol that includes the study of the cardiac conduction system and brainstem in serial sections, as suggested by the national reference center of the national law [45,46]. However, it is important to keep in mind that positive results from various examinations do not always translate into diagnostic findings. In the past, certain observations, such as alveolar hemorrhages and gastric contents in conducting airways, were thought to be diagnostically significant but are no longer considered so. Furthermore, petechial hemorrhages of the intrathoracic organs are frequently reported in cases of unexplained infant deaths. However, they are not diagnostically relevant for identifying a specific cause of death. In the effort to explain these tragic deaths, there is a risk of overinterpreting minor findings as causally connected to death or fixating on “positive” findings that lack known diagnostic significance. The presence of resource deficiency frequently plays a role in the decision-making process of discerning whether or not to further examine findings that may be of questionable significance. This underscores the importance of having a set of fundamental autopsy procedures and supplementary testing as a basic routine for documenting pediatric deaths with no clear explanation. The National Association of Medical Examiners (NAME) has established a minimal level of acceptable performance standards for autopsies. However, forensic autopsy performance standard B4 does not provide any direction for pediatric autopsies. Standard B3.2 does state that an autopsy must be carried out in situations where death in infants and young children is unexpected and unexplained [47,48,49]. Nevertheless, there is no widely accepted definition of what constitutes a “thorough” autopsy for unexplained pediatric deaths. Ensuring that autopsies are conducted in situations of unexpected and unexplained death in infants and young children requires the implementation of standardized systems and universally accepted documentation methods. Due to changes in local policies and procedures, not all infant deaths are investigated or autopsied in a consistent manner at present. To ensure consistency and thoroughness in investigating unexplained pediatric deaths, standardized systems and universally accepted methods are essential. Determining the root cause of sudden infant and child deaths requires proper neuropathology evaluation. Autopsies are most effective in identifying infectious etiology or not-diagnosed congenital heart lesions. Conversely, scene analysis and gross autopsy findings help reveal the cause of death in accidents, neglect/abuse, and homicide. When diagnosing the causes of death due to disease, histology, neuropathology, and microbiology are the most useful tools. In order to increase the diagnostic yield, it is important to have standardized systems and universally accepted methods to document unexplained pediatric deaths.

## 4. Discussion

The comprehensive analysis of autopsy procedures in unexplained pediatric deaths is pivotal in determining the cause of death. Forensic pathologists play a crucial role in this process and are expected to use stringent criteria to shift away from SIDS and undetermined or asphyxia categories. The study highlights the importance of implementing universally accepted operating protocols to identify risk factors and document unexplained pediatric deaths. Moreover, standardized systems and universally accepted methods can provide multiple benefits, including improving international surveillance of similar deaths in children beyond infancy. The study also emphasizes the limitations of pos-mortem diagnosis in cases where the cause of death cannot be determined by traditional autopsy methods. Therefore, it is important to continue developing new and more accurate evidence in determining causation through randomized controlled trials. The study also highlights the need for death scene investigations in cases of SIDS, which can generate new hypotheses concerning risk factors. It concludes by emphasizing the need to address the limitations of imaging very small fetuses and to provide explicit guidance for death certifiers in the use of alternative diagnoses.

### 4.1. Unexplained Pediatric Disorders (UPD): The Need for Standardized Reporting and Global Informatics System

Unexplained pediatric disorders remain a significant challenge for healthcare providers and researchers worldwide. These disorders are characterized by symptoms that cannot be attributed to any known medical condition, and they often result in severe consequences for affected children and their families. Understanding the causes and implications of these disorders is critical to developing effective treatments. However, the lack of standardized reporting and a global informatics system for these disorders has hindered progress in this field.

### 4.2. Need for Standardized Reporting and a Global Informatics System

To ameliorate healthcare reform, UPD Standardized reporting and a global informatics system must be implemented. To enhance data quality, considerable investments in electronic health information systems, tertiary education programs, and data collection systems are required. In areas with limited resources, standardized reporting and a global informatics system are indispensable for efficient data management and analysis [47]. Data quality is critical in drawing appropriate conclusions from health information systems. Aligning indicators with global standards will facilitate the removal of duplicative collection processes. Establishing a single source of information pertaining to indicator definitions, data collection tools, and management processes is imperative in order to ensure a uniform data system across all healthcare programs. By institutionalizing this best practice, the healthcare industry can maintain consistency in its approach to information management. Healthcare necessitates standards for information sharing and interoperability, and to improve healthcare, standardized reporting and a global informatics system are imperative [48].

There are several challenges that need to be addressed before a connected healthcare system can be implemented [49]. First, there is the technical challenge of making sure that the system is able to accurately collect and process data. This can be addressed by hiring more clinical informaticians to design and customize systems [50]. Additionally, systems should be designed to support communication and provide flexibility to better fit real work practices in order for them to be more successful [50].

The research paper highlights the need for standardized reporting and a global informatics system to address unexplained pediatric disorders. The paper concludes that the need for health informatics and related standards requires a comprehensive review of relevant literature and regulations/law documents, and governments and health informatics partners are taking initiatives to solve the problem of lacking standards. Overall, the paper provides valuable insights into the need for a more comprehensive and standardized approach to address unexplained pediatric disorders.

Finally, we propose a universally accepted international computerized platform where all health systems, both small and large, with diversified characteristics (i.e., central and territorial) can use it in order to have a real evaluation of UPD cases and autopsy results with the creation of reports autopsy standards and international universal coding systems. The protocol could be a useful tool to shed more light on a phenomenon that is still unknown despite the development of new technologies.

## Data Availability

Not applicable to this article as no datasets were generated.

## References

[1] Shapiro-Mendoza C.K., Woodworth K.R., Cottengim C.R., Lambert A.B.E., Harvey E.M., Monsour M., Parks S.E., Barfield W.D. (2023). Sudden Unexpected Infant Deaths: 2015–2020. Pediatrics.

[2] Boyer B.T., Lowell G.S., Roehler D.R., Quinlan K.P. (2022). Racial and ethnic disparities of sudden unexpected infant death in large US cities: A descriptive epidemiological study. Inj. Epidemiol..

[3] Krous H.F., Beckwith J.B., Byard R.W., Rognum T.O., Bajanowski T., Corey T., Cutz E., Hanzlick R., Keens T.G., Mitchell E.A. (2004). Sudden Infant Death Syndrome and Unclassified Sudden Infant Deaths: A Definitional and Diagnostic Approach. Pediatrics.

[4] Burns K.M., Cottengim C., Dykstra H., Faulkner M., Lambert A.B.E., MacLeod H., Novak A., Parks S.E., Russell M.W., Shapiro-Mendoza C.K. (2020). Epidemiology of Sudden Death in a Population-Based Study of Infants and Children. J. Pediatr. X.

[5] Leitner D.F., William C., Faustin A., Askenazi M., Kanshin E., Snuderl M., McGuone D., Wisniewski T., Ueberheide B., Gould L. (2022). Proteomic differences in hippocampus and cortex of sudden unexplained death in childhood. Acta Neuropathol..

[6] Vincent A., Chu N.T., Shah A., Avanthika C., Jhaveri S., Singh K., Limaye O.M., Boddu H. (2023). Sudden Infant Death Syndrome: Risk Factors and Newer Risk Reduction Strategies. Cureus.

[7] Brixey S.N., Kopp B.C., Schlotthauer A.E., Collier A., Corden T.E. (2011). Use of child death review to inform sudden unexplained infant deaths occurring in a large urban setting. Inj. Prev..

[8] McGarvey C.M., O’Regan M., Cryan J., Treacy A., Hamilton K., Devaney D., Matthews T. (2012). Sudden unexplained death in childhood (1–4 years) in Ireland: An epidemiological profile and comparison with SIDS. Arch. Dis. Child..

[9] Kinney H.C., Armstrong D.L., Chadwick A.E., Crandall L.A., Hilbert C., Belliveau R.A., Kupsky W.J., Krous H.F. (2007). Sudden Death in Toddlers Associated with Developmental Abnormalities of the Hippocampus: A Report of Five Cases. Pediatr. Dev. Pathol..

[10] Crandall L.G., Lee J.H., Stainman R., Friedman D., Devinsky O. (2019). Potential Role of Febrile Seizures and Other Risk Factors Associated With Sudden Deaths in Children. JAMA Netw. Open.

[11] Hunt C.E., Darnall R.A., McEntire B.L., Hyma B.A. (2015). Assigning cause for sudden unexpected infant death. Forensic Sci. Med. Pathol..

[12] Tabor P.D., Ragan K. (2015). Infant Death Scene Investigation. J. Forensic Nurs..

[13] Pasquale-Styles M.A., Tackitt P.L., Schmidt C.J. (2007). Infant Death Scene Investigation and the Assessment of Potential Risk Factors for Asphyxia: A Review of 209 Sudden Unexpected Infant Deaths. J. Forensic Sci..

[14] Sauber-Schatz E.K., Sappenfield W.M., Shapiro-Mendoza C.K. (2014). Comprehensive Review of Sleep-Related Sudden Unexpected Infant Deaths and Their Investigations: Florida 2008. Matern. Child Health J..

[15] Fitzgerald D.A., Jeffery H., Arbuckle S., du Toit-Prinsloo L., O’Sullivan T., Waters K. (2021). Sudden Unexpected Death in Infancy [SUDI]: What the clinician, pathologist, coroner and researchers want to know. Paediatr. Respir. Rev..

[16] Shapiro-Mendoza C.K., Palusci V.J., Hoffman B., Batra E., Yester M., Corey T.S., Sens M.A., Moon R.Y., Goodstein M.H., Abu Jawdeh E. (2021). Half Century Since SIDS: A Reappraisal of Terminology. Pediatrics.

[17] Palusci V.J., Kay A.J., Batra E., Moon R.Y., Corey T.S., Andrew T., Graham M., Neglect C.O.C.A.A., Prevention S.O.C.D.R.A., Syndrome T.F.O.S.I.D. (2019). Identifying Child Abuse Fatalities During Infancy. Pediatrics.

[18] Pasquale-Styles M.A., Regensburg M., Bao R. (2017). Sudden Unexpected Infant Death Certification in New York City: Intra-Agency Guideline Compliance and Variables that May Influence Death Certification. Acad. Forensic Pathol..

[19] Berkowitz C.D. (2012). Sudden Infant Death Syndrome, Sudden Unexpected Infant Death, and Apparent Life-Threatening Events. Adv. Pediatr..

[20] Mitchell R.A., DiAngelo C., Morgan D. (2017). Medicolegal Death Investigation of Sudden Unexpected Infant Deaths. Pediatr. Ann..

[21] Gilliland M., Luckenbach M.W., Chenier T.C. (1994). Systemic and ocular findings in 169 prospectively studied child deaths: Retinal hemorrhages usually mean child abuse. Forensic Sci. Int..

[22] Moon R.Y., Horne R.S., Hauck F.R. (2007). Sudden infant death syndrome. Lancet.

[23] Lambert A.B.E., Parks S.E., Camperlengo L., Cottengim C., Anderson R.L., Covington T.M., Shapiro-Mendoza C.K. (2016). Death Scene Investigation and Autopsy Practices in Sudden Unexpected Infant Deaths. J. Pediatr..

[24] Shapiro-Mendoza C.K., Parks S.E., Brustrom J., Andrew T., Camperlengo L., Fudenberg J., Payn B., Rhoda D. (2017). Variations in Cause-of-Death Determination for Sudden Unexpected Infant Deaths. Pediatrics.

[25] Bennett T., Martin L.J., Heathfield L.J. (2019). A retrospective study of death scene investigation practices for sudden unexpected death of infants (SUDI) in Cape Town, South Africa. Forensic Sci. Med. Pathol..

[26] Aquila I., Falcone C., Di Nunzio C., Tamburrini O., Boca S., Ricci P. (2013). Virtopsy versus autopsy in unusual case of asphyxia: Case report. Forensic Sci. Int..

[27] Langan Y., Nashef L., Sander J.W.A.S. (2000). Sudden unexpected death in epilepsy: A series of witnessed deaths. J. Neurol. Neurosurg. Psychiatry.

[28] Lathers C.M., Schraeder P.L. (2009). Verbal autopsies and SUDEP. Epilepsy Behav..

[29] Pilmer C.M., Porter B., Kirsh J.A., Hicks A.L., Gledhill N., Jamnik V., Faught B.E., Hildebrandt D., McCartney N., Gow R.M. (2013). Scope and nature of sudden cardiac death before age 40 in Ontario: A report from the Cardiac Death Advisory Committee of the Office of the Chief Coroner. Hear. Rhythm..

[30] RamachandranNair R., Jack S.M., Meaney B.F., Ronen G.M. (2013). SUDEP: What do parents want to know?. Epilepsy Behav..

[31] Aquila I., Sacco M., Gratteri S., Sirianni M., De Fazio P., Ricci P. (2018). The “Social-mobile autopsy”: The evolution of psychological autopsy with new technologies in forensic investigations on suicide. Leg. Med..

[32] Aquila I., Sacco M.A., Ricci C., Gratteri S., Ricci P. (2020). Quarantine of the Covid-19 pandemic in suicide: A psychological autopsy. Med. Leg. J..

[33] Goldstein R.D., Blair P.S., Sens M.A., Shapiro-Mendoza C.K., Krous H.F., Rognum T.O., Moon R.Y., 3rd International Congress on Sudden Infant and Child Death (2019). Inconsistent classification of unexplained sudden deaths in infants and children hinders surveillance, prevention and research: Recommendations from The 3rd International Congress on Sudden Infant and Child Death. Forensic Sci. Med. Pathol..

[34] Speelman A.C., Engel-Hills P.C., Martin L.J., van Rijn R.R., Offiah A.C. (2022). Postmortem computed tomography plus forensic autopsy for determining the cause of death in child fatalities. Pediatr. Radiol..

[35] Tester D.J., Ackerman M.J. (2006). The role of molecular autopsy in unexplained sudden cardiac death. Curr. Opin. Cardiol..

[36] Arthurs O.J., Hutchinson J.C., Sebire N.J. (2017). Current issues in postmortem imaging of perinatal and forensic childhood deaths. Forensic Sci. Med. Pathol..

[37] Gorincour G., Sarda-Quarello L., Laurent P.-E., Brough A., Rutty G.N. (2015). The future of pediatric and perinatal postmortem imaging. Pediatr. Radiol..

[38] Campobasso C.P., Introna F. (2001). The forensic entomologist in the context of the forensic pathologist’s role. Forensic Sci. Int..

[39] Bajanowski T., Vege A., Byard R.W., Krous H.F., Arnestad M., Bachs L., Banner J., Blair P.S., Borthne A., Dettmeyer R. (2007). Sudden infant death syndrome (SIDS)—Standardised investigations and classification: Recommendations. Forensic Sci. Int..

[40] Campuzano O., Allegue C., Partemi S., Iglesias A., Oliva A., Brugada R. (2014). Negative autopsy and sudden cardiac death. Int. J. Leg. Med..

[41] Montanari E., Bonasoni M.P., Licata M., Salomone A., Gerace E., Vivarelli M., Giorgetti R., Tagliabracci A. (2018). Toxicological and histological analyses for a stillborn delivered by a mother under methadone maintenance therapy. Forensic Toxicol..

[42] Montanari E., Bonasoni M.P., Alessandrini F., Frazzi R., Mocchegiani F., Busardò F.P., Giorgetti R., Tagliabracci A. (2019). CYP2B6, ABCB1 and OPRM1 profile in a stillborn affected by chronic methadone intoxication. Forensic Toxicol..

[43] Ottaviani G., Ramos S.G. (2023). Autopsy for Medical Diagnostics: Finding the Cause of Sudden Unexpected Death through Investigation of the Cardiac Conduction System by Serial Sections. Diagnostics.

[44] Knight L.D., Andrew T.A., Eason E.A., Landi K., Lear K.C., McCleskey B., Pinneri K., Bundock E.A., Corey T.S., Andrew T.A., Crandall L.G., Eason E.A., Gunther W.M., Moon R.Y., Palusci V.J., Schmidt C.M., Sens M.A. (2019). Autopsy. Unexplained Pediatric Deaths: Investigation, Certification, and Family Needs.

[45] Matturri L., Ottaviani G., Lavezzi A.M. (2008). Guidelines for neuropathologic diagnostics of perinatal unexpected loss and sudden infant death syndrome (SIDS)—A technical protocol. Virchows Arch..

[46] Matturri L., Ottaviani G., Lavezzi A.M. (2005). Techniques and Criteria in Pathologic and Forensic-Medical Diagnostics in Sudden Unexpected Infant and Perinatal Death. Am. J. Clin. Pathol..

[47] Ledikwe J.H., Grignon J., Lebelonyane R., Ludick S., Matshediso E., Sento B.W., Sharma A., Semo B.-W. (2014). Improving the quality of health information: A qualitative assessment of data management and reporting systems in Botswana. Health Res. Policy Syst..

[48] Alvarez R.C., Zelmer J. (1998). Standardization in health informatics in Canada. Int. J. Med. Inform..

[49] Riley C., Xie B., Khurshid A. (2021). Challenges encountered in comparing international policy responses to COVID-19 and their effects. Health Res. Policy Syst..

[50] Zeadally S., Siddiqui F., Baig Z., Ibrahim A. (2020). Smart healthcare: Challenges and potential solutions using internet of things (IoT) and big data analytics. PSU Res. Rev..

